# A Comparison of Three Common Rehabilitation Interventions Used to Improve Cardiovascular Fitness after Stroke: An Overview of the Literature

**DOI:** 10.1155/2023/4350851

**Published:** 2023-04-11

**Authors:** Salem F. Alatawi

**Affiliations:** Department of Physical Therapy, Faculty of Applied Medical Sciences, University of Tabuk, Tabuk City, Saudi Arabia

## Abstract

**Background:**

One of the most frequent consequences of stroke is a reduction in heart function. After a stroke, one of the main aims of physiotherapy practice is to improve cardiovascular fitness (CVF). This paper is aimed at identifying the best effective intervention of improving the cardiovascular fitness (CVF) after stroke while focusing on body weight-supported treadmill training (BWSTT), over gait training (OGT), and therapeutic exercise.

**Methods:**

Different electronic databases were searched until July 2022. Controlled randomized trials examining the effects of BWSTT, OGT, and therapeutic exercise to improve CVF on an ambulatory person with stroke, written in English and reporting cardiovascular fitness or at least one of its indicators, such as peak oxygen consumption (VO_2_), gait speed, gait energy expenditure, and functional independence measure for locomotion (FIM-L), were included. The quality of the methodology was evaluated using the Physiotherapy Evidence Database (PEDro) scale.

**Results:**

The research yielded 3854 relevant studies, of which 22 met the eligibility criteria. The primary indicators of the CVF, VO_2_ and energy expenditure, were used to examine the CVF in only three studies, while the rest used other indicators of the CVF. There was a lack of sufficient evidence to establish the superiority of one intervention over another. However, it appears that utilizing BWSTT to improve the CVF after stroke is effective.

**Conclusion:**

Physiotherapy has the potential to enhance the CVF of stroke patients. However, effective interventions and long-term effects remain debatable.

## 1. Introduction

A stroke may cause damage to the nervous system and restrict movement, limiting motor function and physical activity [1]. Low physical activity causes vascular impairments in stroke patients, including reduced lung diffusion capacity owing to partly paralyzed muscles, chest wall, and diaphragm movement, ventilation-perfusion inconsistency, or partial respiratory failure due to reduced lung volume [2–4]. However, the research cannot provide a definitive and precise explanation for the causes of cardiovascular impairment after a stroke. In stroke patients, poor respiratory outcomes are associated with chronic obstructive pulmonary disease (COPD), cardiovascular dysfunction, smoking, and inactivity [5].

Cardiovascular fitness (CVF) is the capacity of the circulatory and respiratory systems to deliver oxygen to the skeletal muscles during exercise [6]. A decrease in chest wall motion may lead to secondary muscular fibrosis in the ribs, which further inhibits inspiration owing to a decrease in maximum inspiration pressure [7]. After a stroke, CVF is diminished, with VO_2_ peak values ranging from 8 to 22 mL/kg/min or 26 to 87 percent, respectively, of those of healthy individuals of the same age and gender [8].

The rehabilitation process after a stroke is challenging and has been connected to a wide range of physiological and psychological long-term consequences [9]. Patients and carers must participate in the rehabilitation process after a stroke as members of multidisciplinary teams. Multidisciplinary medical professionals, such as physiotherapists, nurses, occupational therapists, speech therapists, psychologists, and nutritionists, may comprise these teams [10, 11].

Several studies [12–15] have discovered a correlation between the patients' poststroke cardiovascular (CV) health in terms of peak oxygen consumption (VO_2_), energy expenditure, the patients' diminished ability to regain their previous level of independence, walking ability, and gait speed. The present study utilized previous findings to identify the most commonly used indicators of improvement in cardiovascular fitness. Peak oxygen consumption and total energy expenditure appeared to be significant indicators of cardiovascular fitness. Additionally, gait speed, activities of daily living, and walking ability revealed enhanced gait performance, which eventually improved cardiovascular fitness [11–15]. Consequently, based on these understandings, this paper acknowledges the necessity of discovering a safe and effective physiotherapy intervention for improving poststroke patients' cardiovascular fitness and gait performance.

For poststroke patients, body weight-supported treadmill training (BWSTT), over gait training (OGT), and therapeutic exercises were the three most prominent forms of physiotherapy interventions [12–16].

BWSTT employs overhead suspension to support a patient's weight while walking on a treadmill. The procedure symmetrically removes weight from the lower extremities [17]. BWSTT may be employed early in poststroke rehabilitation when patients are unable to properly bear their own weight [5.14]. BWSTT therapeutic benefits include a focus on dynamic and repetitive task-specific exercise, as well as weight bearing, balance, and stepping [17]. However, this technique does not overcompensate for the patients' preserved motor capabilities after a stroke [15]. Additionally, since this intervention requires 2-3 staff members, it may be too expensive for many treatment centers [15]. In a systematic review focused on BWSTT, the findings showed that there was improvement in the walking speed of poststroke patients following the BWSTT intervention. However, the review was unable to find data supporting the advantages of BWSTT over other physiotherapy interventions such as OGT and therapeutic exercises [18]. In a 2017 Cochrane review, Mehrholz et al. [19] observed that treadmill training increased walking speed and endurance for stroke patients who could walk independently, regardless of body weight support. However, the improvements in walking speed and endurance were not maintained over time.

Overground training involves the physiotherapist observing and guiding the patient's walking movements while they are doing ground exercises [13]. It does not need either support from the body's weight or functional electrical stimulation [13]. In contrast to the BWSTT, which needs 2-3 staff members to complete, the relevant exercises are designed to improve the patient's gait function and are performed by the patient [15]. OGT is cost-effective and adaptable to any setting since it is based on core physiotherapy skills such as observation, encouragement, and instruction in exercises to improve gait function [12]. However, the evidence for the OGT's usefulness in improving poststroke gait function is mixed [13]. In a previous systematic review that examined the impact of OGT on gait functions, it was concluded that there was insufficient evidence to support the advantages of OGT on gait functions [20]. However, there is a knowledge gap regarding the efficacy of OGT in improving CVF when compared to other physiotherapy interventions such as BWSTT and therapeutic exercises.

Therapeutic exercise is critical and one of the most commonly used forms of physiotherapy rehabilitation for reducing the risk of future cardiovascular events and recurrent stroke following a stroke [21]. Therapeutic exercise is exercise that has been prescribed for the purpose of treating impairments, improving muscular and skeletal function, and/or maintaining a state of health [22]. Strength activities, endurance exercises that engage large muscle groups to increase cardiovascular endurance, flexibility exercises achieved via stretching and mobility, and balance and coordination exercises are the most common forms of exercise [21, 22]. Research has shown that improving cardiac breathing may improve cardiopulmonary function. The circulation of people who have had a stroke may benefit from exercise. Increasing heart rate and breathing rate via aerobic exercise is beneficial [22–24]. Previous systematic reviews showed that exercises may improve VO_2_ peak and 6MWT in stroke survivors [6], increase aerobic capacity and walking performance [21], and improve poststroke patients' activities of daily living (ADL) [25].

The above interventions (BWSTT, OGT, and therapeutic exercises) were previously widely used in physiotherapy practices to enhance patients' cardiovascular health and avoid recurrence of stroke and other cardiac problems [12, 16, 20, 21]. However, there is a paucity of information on the role of physiotherapy in increasing cardiovascular fitness after stroke, and no study has been conducted to investigate the efficacy of these routinely used physiotherapy approaches (BWSTT, OGT, and therapeutic exercises). As a result, the aim of this paper was to determine the best effective intervention for improving CVF in ambulatory poststroke patients, with a focus on BWSTT, OGT, and therapeutic activities.

## 2. Methods

### 2.1. Search Strategy

**Figure 1:**
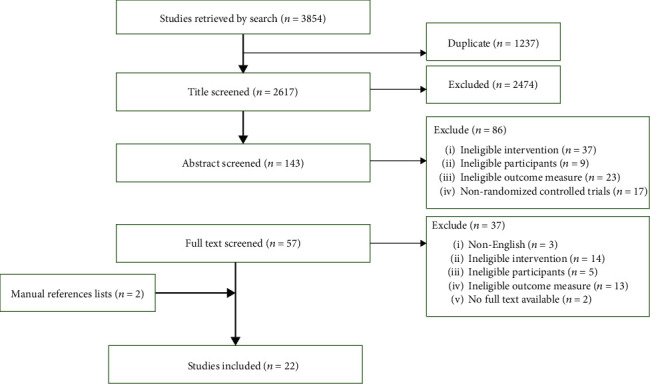
Screening for the inclusion of studies.

The different electronic databases such as PubMed, AMED, EBSCO, Embase, MEDLINE, CINAHL, and Web of Science were systematically searched from their inception to July 28, 2022. The search strategy used included a combination of the following medical subject heading (MeSH) terms: “stroke rehabilitation” and “cardiovascular fitness.” These were combined with the following free-text terms: “VO2,” “gait speed,” “gait energy expenditure,” and “functional independence measure for locomotion (FIM-L)” (Figure 1). Additionally, a manual review of the reference lists of all included studies was conducted to see if any new study satisfied the inclusion criteria.

### 2.2. Inclusion and Exclusion Criteria

The following criteria were used to choose publications for this paper: (1) randomized controlled trials; (2) ambulatory stroke patients; (3) administration of any of the interventions (BWSTT, OGT, or therapeutic exercise); and (4) evaluation of the intervention's effects using the VO_2_, gait energy expenditure, gait speed, or FIM-L or a combination of these outcome measures. Studies were excluded if they (1) were published in a language other than English or (2) involved treadmill training without BWSTT or (3) examined interventions other than BWSTT, OGT, and therapeutic exercise.

### 2.3. Data Extraction

All data were extracted from papers that met the inclusion criteria. The following data was extracted from the original reports: (1) authors, year of publication, and country; (2) research aim; (3) study design and sample size; (4) outcome measures; (5) findings; and (6) conclusion.

### 2.4. The Quality of the Included Papers

The Physiotherapy Evidence Database (PEDro) scale was used to evaluate the methodological quality of the included randomized controlled studies [26]. The website (https://www.pedro.org.au/) provided available methodological quality ratings. The scale consists of 10 items, with a score of 0 indicating “no” and 1 indicating “yes.” Both a score of five out of ten and a score of six out of ten have been used as a cutoff score for good quality [27, 28]. However, since blinded participants and therapists are almost impossible to find in research examining the effects of physical activities and the highest attainable score is eight out of ten, a score of five out of ten was utilized as the cutoff [28]. Therefore, a score of 5 or higher was considered “high quality,” while a score of 4 or less was “low quality” in this study (Table 1).

## 3. Results

### 3.1. The Characteristics of the Included Studies

The electronic searches yielded 3854 studies, of which 20 met the inclusion criteria. Figure 1 shows the suitable screening procedure and the reasons for exclusions. A thorough check of the reference lists of the included papers uncovered two more studies. In total, 22 papers were included in this paper.  Table 2 provides a summary of all the included studies. The sample sizes of the included studies ranged from 12 [49] to 408 [36]. There were a total of 1591 participants distributed over the studies, with an average of 72 participants per study. Four studies recruited individuals with an acute stroke; nine studies recruited subacute stroke patients, and nine studies recruited chronic stroke patients.

### 3.2. The Quality of the Included Studies

Five studies received a score of 8 out of 10, four studies received a score of 7 out of 10, and six studies received a score of 6 out of 10 on the PEDro scale. Subjects and therapists are blinded to PEDro scale items that were not fulfilled in all studies. However, it looks challenging to blind participants and therapists in interventions consisting of exercise programs [27]. Nevertheless, the majority of studies met the criteria pertaining to random allocation, point measurements and variability, and baseline subject similarity. These elements give insight into the strength of the subject recruitment procedure and indicate that the data obtained from the participants were meaningfully analyzed. In a number of studies, the concealed allocation and the analysis of the intention to treat were absent. In addition, the sample sizes of the majority of the reviewed studies were small. Furthermore, the follow-up durations in the included research were rather short, with the majority of studies examining the efficacy of physiotherapy immediately after the completion of the interventions. Despite these limitations, the included papers reflect the best available research on the efficacy of BWSTT, OGT, and therapeutic exercise in restoring poststroke patients' cardiovascular fitness and gait performance. In addition, a variety of interventions pertaining to the significance of physiotherapy in improving cardiovascular fitness after stroke are covered in these studies. This paper may provide a comprehensive description of the most efficient physiotherapy intervention for improving CVF after a stroke.

### 3.3. Physiotherapy Interventions on the CVF after Stroke

This paper comprised studies that explored the comparative efficacy of three different types of physiotherapy interventions: BWSTT, OGT, and therapeutic exercise. These interventions were aimed at restoring cardiovascular fitness and gait performance in poststroke patients.

Twenty-two studies [29–50] looked at whether or not these interventions were helpful on their own or in combination with one another. However, despite the fact that the goal of each of these included studies was to increase patients' cardiovascular fitness after a stroke, the end indicators that were utilized to determine whether they were successful in accomplishing this objective differed from study to study. As a result of these data, it is plainly obvious that the indications that are most often employed as markers to promote cardiovascular fitness are VO_2_, energy expenditure, walking speed, gait speed, activities of daily living, and walking ability.

#### 3.3.1. Peak Oxygen Consumption and Energy Expenditure

The major outcomes of cardiovascular fitness to look at when evaluating the effectiveness of physiotherapy interventions in increasing cardiovascular fitness in poststroke patients are VO_2_ and energy consumption. This is due to the fact that these outcomes serve as indicators of cardiovascular fitness.

Three studies used VO_2_ and energy expenditure as outcome measures to examine the effectiveness of BWSTT in improving VO_2_ in poststroke patients compared to conventional treatment [35, 43, 49]. The results of the three studies indicated that BWSTT is more efficient in increasing the cardiovascular fitness of stroke patients. On the PEDro scale, the three studies scored 8, 5, and 4, respectively [35, 43, 49].

#### 3.3.2. Walking Speed/Gait Speed

The patient's walking speed, or “gait speed,” is a secondary measure of cardiovascular health after a stroke. Several studies have investigated the effects of BWSTT alone [42], in combination with OGT [30, 44], or in conjunction with locomotor training [41, 46] on walking speed/gait speed in ambulatory stroke patients. Sullivan et al. [42] revealed that BWSTT increases walking speed in ambulatory stroke patients more successfully than cycling with resistance. Other findings showed that the combination of BWSTT and OGT is more effective in improving poststroke patients' walking speed [30, 44]. Other studies have shown that BWSTT with locomotor training (robot-assisted treadmill training) is superior to conventional therapy for increasing walking speed [41, 45]. Others, however, have shown no statistically significant differences between BWSTT and OGT in terms of enhancing the walking speed of stroke patients [29, 31, 32, 36, 50]. Various studies have shown that OGT [33] and conventional therapy [40] are superior to BWSTT for increasing walking speed. With the exception of one study [29], almost all of the studies in this section scored above 5 on the PEDro scale; however, the evidence is equivocal as to whether these interventions may be beneficial in improving walking speed in poststroke patients.

#### 3.3.3. Activities of Daily Living (ADL)

Cardiovascular fitness may have an effect on a person's level of functional independence, as well as their motor, cognitive, and ADL skills. Some studies evaluated the CVF of poststroke patients using secondary outcomes such as functional independence, motor and cognitive abilities, and ADL. These studies show that BWSTT improves the motor and functional outcomes of people who have had a stroke, either alone [35, 37, 41, 47] or in combination with other interventions like FES [34], OGT [44], and therapeutic exercise [38]. However, other studies indicate that BWSTT is not better than OGT or therapeutic exercise interventions [24, 30–32, 36, 39, 40, 42, 47, 49, 50]. All the studies included in this section were of excellent quality, since their PEDro scores were at least 5, except one study [45].

#### 3.3.4. Walking Abilities

Stroke patients' cardiovascular fitness may be determined by their ability to walk. Some researchers discovered that the BWSTT had a significant impact on the walking and functional ability of poststroke patients, whether it was used on its own [31, 41, 45, 47, 48], combined with functional electrical stimulation (FES) [34], combined with locomotor training [46], or combined with functional conventional rehabilitation [37]. On the other hand, several studies discovered that BWSTT had no meaningful influence [32, 33, 36, 39]. Even though the majority of these studies had high PEDro scores, there was not enough data to compare the effectiveness of BWSTT to other interventions.

## 4. Discussion

In the practice of physiotherapy for poststroke patients, it was vital to achieve a safe and effective CVF-improving rehabilitation approach. This study is aimed at comparing the effectiveness of BWSTT, OGT, and therapeutic exercise in improving CVF in stroke patients.

Using the PEDro scale, the included studies were evaluated critically to show that the paper was of high quality. With the exception of three studies [29, 45, 49], all research studies received a score of 5 or above. Due to the paucity of studies on the subject, however, these low-scoring studies were included in this paper. Even so, the fact that there is not enough relevant research will not necessarily stop this evaluation from coming up with useful results.

In order to determine which of these physiotherapy interventions is preferable for the rehabilitation process, it was necessary to concentrate on certain cardiovascular health indicators in poststroke patients. So, this paper focused on important measures of cardiovascular health in stroke patients, like VO_2_, energy expenditure, gait speed, walking ability, and functional independence related to ADL [12–15].

The principal results of the included studies identified the primary indicators that might assist physiotherapists in enhancing CVF throughout the rehabilitation process. Peak oxygen consumption and energy expenditure were identified as the primary outcome measures for improving CVF in rehabilitation practice. In the same way, other indicators of gait performance like gait speed, walking ability, and activities of daily living (ADL) were found to be important indicators of gait performance that may show a change in CVF in stroke patients.

This study's findings indicate that BWSTT is effective in improving the major cardiovascular fitness measures of peak oxygen use and energy expenditure [35, 43, 49]. However, the evaluation identified a few trials that focused only on these adverse effects of CV fitness, and the included research did not compare BWSTT to other interventions.

BWSTT has shown benefits for improving CVF in stroke patients. Three studies [35, 43, 49] indicate that BWSTT may improve poststroke patients' major cardiovascular fitness indicators, VO_2_ and energy expenditure. Due to the lack of comparisons with OGT and therapeutic exercise, it is not possible to conclude with certainty that BWSTT is the most effective physiotherapy intervention for increasing VO_2_ and energy expenditure in poststroke patients. The fact that one of these studies received a score of eight out of ten on the PEDro scale [35], while the others [43, 49] had scores of five and four, respectively, implies that the robustness of these studies differs. Consistent with this finding, prior reviews have shown that treadmill exercises may enhance VO_2_ and 6MWT in stroke patients [6, 21].

Similarly, the included studies [30, 41, 42, 44, 46] demonstrate that BWSST improves walking speed, which is considered a secondary indicator for improvement in CVF, among stroke patients, regardless of whether it is used alone or in conjunction with other interventions. However, other studies that compared the efficacy of BWSTT and OGT interventions for increasing walking speed after a stroke did not identify any advantage that could be attributable to one intervention being superior to the other. In addition, other included studies revealed that OGT intervention and conventional therapy (including therapeutic exercise) were superior to BWSTT when compared separately [33, 40]. This paper cannot declare with certainty that BWSTT is the most effective physiotherapy intervention for enhancing walking speed in poststroke patients. The findings of the present study are consistent with those of previous reviews [18, 19] that indicated the benefits of BWSTT in improving walking speed and endurance among stroke patients. However, these reviews lack comparison with the OGT and therapeutic exercises.

The last two indicators that were found were ADL and walking abilities, both of which addressed the patients' ability for functional independence after a stroke. The majority of the included studies in this part had equivocal results, making it challenging for the researcher to identify the most effective physiotherapy interventions. The included studies [25, 27, 41, 47] indicate that BWSTT is helpful for enhancing ADL. Other research [34, 37, 46] suggests that BWSTT paired with other interventions is more effective in enhancing ADL. On the other hand, several studies [24, 30–32, 36, 39, 40, 42, 47, 49, 50] did not demonstrate a substantial benefit for BWSTT in terms of ADL improvement or when compared to other interventions. Similarity aside, the included studies have shown that BWSTT is beneficial in enhancing the walking ability of stroke patients, whether administered alone [31, 41, 45, 47] or in combination with other interventions [34, 37, 46]. However, other studies [32, 33, 36, 39] have similarly shown that the advantage of BWSTT over other interventions cannot be demonstrated. Again, due to a paucity of evidence, it is hard to draw solid conclusions about the advantages of BWSTT in comparison to other interventions. In agreement with these results, a previous systematic review [20] concluded that there was insufficient evidence to establish the efficacy of OGT on gait functions.

Several limitations should be acknowledged when interpreting this paper's findings. First, the heterogeneity of the included studies and the variation in outcome measurement parameters between studies, from the primary outcomes of CVF, such as peak oxygen consumption and energy expenditure, to the secondary outcomes, such as gait speed and functional independence, prevent meta-analysis and the generation of specific findings. Second, the majority of included studies examined outcome indicators shortly after intervention. Few studies have included follow-up assessments. However, many studies had short follow-up periods. Therefore, this paper cannot draw a conclusion about the long-term effect of BWSTT, OGT, and therapeutic exercise interventions on the improvement of CVF after stroke. Lastly, the author made both the decision to score the studies using the PEDro scale and the decision to include or exclude the studies independently. However, inclusion and exclusion criteria were mentioned explicitly. Additionally, there was no difference between the PEDro score for each study and the original score provided by the PEDro website.

## 5. Conclusion

The findings of this paper were inadequate to fulfil the research aim of determining the superiority of BWSTT, OGT, and therapeutic exercise in enhancing the CVF in stroke patients. However, the results demonstrated that BWSTT might be more successful in improving both the main and secondary indicators of CVF. It is also possible to conclude that BWSTT is less viable than the other two physiotherapy interventions owing to its high cost and resource requirements. The implications of this paper are thus limited to physiotherapy clinics and large-scale rehabilitation facilities, while alternative settings, such as home care, primary care, and small outpatient clinics, may lack the necessary resources and expertise. It is suggested that more high-quality studies be done in the future to compare the benefits of BWSTT, OGT, and therapeutic activities and to find the best way to improve CVF in stroke patients.

## Figures and Tables

**Table 1:** Summary of the methodological quality of the included studies based on the PEDro scale (*n* = 22).

	Random allocation	Concealed allocation	Baseline similar	Subject blinding	Therapist blinding	Assessor blinding	Adequate follow-up	Intention-to-treat analysis	Between-group comparison	Point estimate	Total
Lura et al. [29]	Yes	No	No	No	No	No	Yes	No	Yes	Yes	4
Gama et al. [30]	Yes	No	Yes	No	No	No	Yes	Yes	Yes	Yes	6
De Paul et al. [31]	Yes	Yes	Yes	No	No	Yes	Yes	Yes	Yes	Yes	8
Middleton et al. [32]	Yes	No	Yes	No	No	Yes	No	Yes	Yes	Yes	6
Combs-Miller et al. [33]	Yes	Yes	Yes	Yes	No	No	Yes	Yes	Yes	Yes	8
Lee et al. [34]	Yes	No	Yes	No	No	Yes	Yes	No	Yes	Yes	6
Mackay-Lyons et al. [35]	Yes	Yes	Yes	No	No	Yes	Yes	Yes	Yes	Yes	8
Duncan et al. [36]	Yes	No	Yes	No	No	Yes	Yes	Yes	Yes	Yes	7
Dean et al. [37]	Yes	Yes	Yes	No	No	Yes	Yes	Yes	Yes	Yes	8
Schwartz et al. [38]	Yes	No	Yes	No	No	Yes	No	Yes	Yes	Yes	6
Franceschini et al. [39]	Yes	No	Yes	No	No	Yes	No	Yes	Yes	Yes	6
Hidler et al. [40]	Yes	No	Yes	No	No	No	Yes	No	Yes	Yes	5
Mayr et al. [41]	Yes	No	No	No	No	Yes	Yes	No	Yes	Yes	5
Sullivan et al. [42]	Yes	No	Yes	No	No	Yes	Yes	Yes	Yes	Yes	7
Macko et al. [43]	Yes	No	Yes	No	No	Yes	No	No	Yes	Yes	5
Ada et al. [44]	Yes	Yes	Yes	No	No	Yes	Yes	No	Yes	Yes	7
Barbeau and Visintin [45]	Yes	No	Yes	No	No	Yes	No	No	Yes	No	4
Duncan et al. [46]	Yes	Yes	Yes	Yes	No	No	Yes	Yes	Yes	Yes	8
Pohl et al. [47]	Yes	No	Yes	No	No	Yes	Yes	No	Yes	Yes	6
Laufer et al. [48]	No	No	Yes	No	No	Yes	Yes	No	Yes	Yes	5
De Cunha Filho et al. [49]	Yes	No	Yes	No	No	No	No	No	Yes	Yes	4
Nilsson et al. [50]	Yes	Yes	Yes	No	No	Yes	Yes	No	Yes	Yes	7

**(a):** 

Study	Aim	Study design	Outcome measures	Findings	Conclusion
Lura et al. [29] USA	To determine the treatment effectiveness of BWSTT against CT in acute poststroke rehabilitation	RCT (*n* = 40) Exp (*n* = 20): BWSTT Con (*n* = 20): CT	Gait outcome (stride length, step width, step asymmetry, and gait speed) 48 hours after discharge, participants' gait was measured using a Qualisys device	(i) Gait outcome (+)	BWSTT seems to have comparable therapeutic effectiveness to conventional overground treatment.
Gama et al. [30] UK	To compare the effects of BWSTT with OGT in chronic stroke patients	RCT (*n* = 28) Exp (*n* = 14): BWSTT Con (*n* = 14): OGT 3x/week for 6 weeks	Gait speed (10MWT) Endurance (6MWT) FIM Measurements were taken before, after, and six weeks after training	(i) 10MWT (+) (ii) 6MWT (+) (iii) FIM (+)	Chronic stroke patients can benefit from 18 sessions of BWSTT or OGT.
De Paul et al. [31] Canada	To compare the effects of an OGT program with BWSTT in chronic stroke patients	RCT (*n* = 71) Exp (*n* = 35): OGT Con (*n* = 36): BWSTT 15 sessions/5 weeks; 1 hour/session	Gait speed FAC Functional balance test (FBT) Assessment: 1 week before training, 1 week after training, and 2 months after training	(i) Gait speed (−) (ii) FAC (−) (iii) FBT (−)	On average, a 15-session program of variable OGT was not better to a BWSTT program of identical frequency, length, and step activity.
Middleton et al. [32] USA	To see whether BWSTT combined with intense mobility training improves gait, balance, and mobility in chronic stroke patients compared to OGT alone	RCT (*n* = 43) Exp (*n* = 23): BWSTT Con (*n* = 20): OGT 10 weeks of 1 hour of gait training (30 hours)	Fast walking speed BBS DGI ABC TUG Fugl-Meyer (FM) Assessment: baseline, after intervention, and 3 months	(i) Walking speed (+) (ii) BBS (+) (iii) DGI (+) (iv) ABC (+) (v) TUG (+) (vi) FM (+)	There were no significant changes between groups.
Combs-Miller et al. [33] USA	To evaluate the benefits of BWSTT and OGT on increasing walking function, activity, and involvement after chronic stroke	RCT (*n* = 20) Exp (*n* = 10): BWSTT Con (*n* = 10): OGT 30 min/5x/week for 2 weeks	Walking speed (10-meter walk) Walking endurance (6-minute walk) Assessment: before, after intervention, 3 months	(i) Walking speed (+) (ii) Walking endurance (+)	OGT outperformed BWSTT in increasing self-selected walking speed.
Lee et al. [34] Korea	The efficacy of BWSTT with FES on functional mobility and gait in chronic stroke patients	RCT (*n* = 30) Exp (*n* = 15): BWSTT and FES for four weeks Con (*n* = 15): BWSTT for 30 minutes, 5 days a week for 4 weeks	Functional movement (BBS, TUG, and the stroke rehabilitation assessment of movement) Gait ability Assessment: baseline, 3 days after the end	(i) BBS (+) (ii) TUG (+) (iii) Stroke rehabilitation assessment of movement (+) (iv) Gait ability (+)	When combined with BWSTT, FES may be a useful therapy for improving stroke patients' functional mobility and gait ability.

BWSTT: body weight-supported treadmill training; OGT: over gait training; CT: conventional treatment; RCT: randomized controlled trial; Exp: experimental group; Con: control group; FES: functional electrical stimulation; FIM: functional independence measure; FAC: functional ambulation category; BBS: Berg balance scale; DGI: dynamic gait index; ABC: activities-specific balance confidence; TUG: timed up and go; FM: Fugl-Meyer.

**(b):** 

Study	Aim	Study design	Outcome measures	Findings	Conclusion
MacKay-Lyons et al. [35] Canada	To compare BWSTT to usual care (UC) in increasing cardiovascular fitness and walking in subacute stroke patients	RCT (*n* = 50) Exp (*n* = 24) BWSTT + UC Con (*n* = 26): UC Assessment: baseline, posttraining, six- and 12-month follow-up, 60 min/5x/week/6 weeks	Peak VO_2_ Six-minute walk test Overground walking speed BBS Assessment: baseline, posttraining, 6-, 12-month follow-up	(i) Peak VO_2_ (+) (ii) Six-minute walk test (+) (iii) Overground walking speed (+) (iv) BBS (+)	BWSTT improves cardiovascular fitness and walking endurance more than UC.
Duncan et al. [36] UK	Determine the impact of locomotor training, including BWSTT, on poststroke walking ability in subacute stroke patients	RCT (*n* = 408) Group 1 (*n* = 139): early locomotor training on a treadmill with BWSTT Group 2 (*n* = 143): late locomotor training (6 months after the stroke) Group 3 (*n* = 126): home exercise program, 2 months poststroke 90 min/12-16 weeks/36 sessions	Functional walking ability Assessment: baseline, 6 months, 12 months	(i) Functional walking ability (−)	Locomotor training, which includes the use of BWSTT, was not shown to be better to progressive exercise at home supervised by a physical therapist.
Dean et al. [37] Australia	Is using a treadmill with BWSTT during inpatient rehabilitation worse than assisted OGT for acute stroke patients?	RCT (*n* = 126) Exp (*n* = 64): BWSTT + usual rehabilitation Con (*n* = 62): OGT + usual rehabilitation 30 minutes/per day/6 months	Walking quality, walking capacity, walking ability Assessment: 6 months following study enrollment	(i) Walking quality (+) (ii) Walking capacity (+) (iii) Walking ability (+)	Six months after starting training, treadmill walking with BWSTT leads to increased walking capacity and perception of walking ability than OGT.
Schwartz et al. [38] Israel	To assess the impact of early and prolonged locomotor therapy with a robotic-assisted gait training (RAGT) system on functional results in subacute stroke patients	RCT (*n* = 67) Exp (*n* = 37): RAGT + regular gait training 30 min/session/day/3 times/6 weeks Con (*n* = 30): gait training 60 min/day/3x/6 weeks	Ability to walk independently (functional ambulatory capacity scale) FMS Gait parameters Assessment: baseline, at 6 weeks	(i) Functional ambulatory capacity scale (+) (ii) FMA (+) (iii) Gait parameters (+)	RAGT combined with regular physiotherapy improved functional and motor results in subacute stroke patients compared to regular physiotherapy alone.
Franceschini et al. [39] Italy	To compare the effectiveness of BWSTT to conventional gait training in people who were unable to walk after a subacute stroke	RCT (*n* = 97) Exp (*n* = 52): CT + BWSTT Con (*n* = 45): CT + OGT Dosage: 60 min/session/per day/for 4 weeks	Motricity index Barthel index FAC 10-meter and 6-minute walk tests Walking handicap scale Assessment: baseline, after 20 sessions, 2 weeks after interventions, and 6 months	(i) Motricity index (+) (ii) Trunk control test Barthel index(+) (iii) FAC (+) (iv) 10-meter and 6-minute walk tests (+) (v) Walking handicap scale (+)	The experimental group's results were not superior to those achieved by conventional treatment.

BWSTT: body weight-supported treadmill training; OGT: over gait training; RCT: randomized controlled trial; Exp: experimental group; Con: control group; BBS: Berg balance scale; TUG: timed up and go; peak VO_2_: peak oxygen consumption; FAC: functional ambulation categories; FMS: functional motor assessment.

**(c):** 

Study	Aim	Study design	Outcome measures	Findings	Conclusion
Hidler et al. [40] USA	To compare the efficacy of BWSTT to conventional gait training in patients with subacute stroke	RCT (*n* = 63) Exp (*n* = 33): BWSTT Con (*n* = 30): CT	Gait assessment cadence (steps/min) Motor assessment scale (MAS) Functional ambulation category BBT Rivermead Assessment: baseline, after 12 and 24 sessions, at 3 months	(i) Gait assessment cadence (steps/min) (+) (ii) MAS (−) (iii) Functional ambulation category (−) (iv) BBT (−) (v) Rivermead (−)	Conventional treatment is more successful than BWSTT in increasing walking speed and endurance.
Mayr et al. [41] Australia	Assess the potential effectiveness of using a BWSTT Lokomat for treadmill training in subacute stroke patients	RCT (*n* = 16) Exp (*n* = 8) = 3 weeks of BWSTT Con (*n* = 8) = = 3 weeks of conventional physical therapy For 9 weeks of treatment	EU-walking scale, Rivermead motor assessment scale, 10 m timed walking speed, 6-minute timed walking distance, motricity index, Ashworth scale of tone Assessment: baseline, at the end of treatment	(i) EU-walking scale (+) (ii) Rivermead motor assessment scale (+) (iii) 10 m timed walking speed (+) (iv) 6-minute timed walking distance (+) (v) Motricity index (+)	BWSTT improved walking performance over the conventional phase.
Sullivan et al. [42] USA	To determine the effect of task-specific and lower-extremity (LE) strength training on walking ability in chronic stroke patients	RCT (*n* = 80) Exp 1 (*n* = 20): BWSTT/UE-Ex group Exp 2 (*n* = 20): BWSTT/UE-Ex group Exp 3 (*n* = 20): BWSTT/CYCLE Exp 4 (*n* = 20): BWSTT/LE-EX	Gait outcomes 10 m comfortable gait speed 10 m fast gait speed 6 m walk distance Assessment: baseline, after 12 sessions, after 24 months, and 6 months of follow-up	Gait outcomes 10 m comfortable gait speed (+) 10 m fast gait speed (+) 6 m walk distance (+)	Daily LE strength training with BWSTT walking did not improve walking outcomes.
Macko et al. [43] USA	To see if BWSTT was more successful than usual treatment in improving ambulatory function and cardiovascular fitness in chronic stroke patients	RCT (*n* = 61) Exp (*n* = 32): BWSTT Con (*n* = 29): usual care 3x/week/40 min/6 months on the treadmill exercise	Cardiovascular fitness: VO_2_ Ambulatory performance capacity (timed-walk performance) Functional mobility (walking improvement questionnaire (WIQ)) Assessment: at baseline, 3 months, and 6 months	(i) VO_2_ (+) (ii) Timed-walk performance (−) (iii) WIQ (+)	BWSTT increases functional mobility and cardiovascular fitness in chronic stroke patients better than conventional rehabilitation.
Ada et al. [44] Australia	To determine the effectiveness of BWSTT and an OGT in reducing disability caused by poor walking after chronic stroke	RCT (*n* = 29) Exp (*n* = 15): BWSTT + OGT Dosage: 30 min/3 times/per week, for 4 weeks Con (*n* = 14): placebo that included a low-intensity home exercise program and routine telephone interaction	Walking speed (over 10 m) Walking capacity (distance over 6 min) Assessment: baseline, 4 weeks, 3 months	(i) Walking speed (+) (ii) Walking capacity (+)	Stroke survivors' walking abilities increased as a result of the BWSTT and OGT.

BWSTT: body weight-supported treadmill training; OGT: over gait training; CT: conventional treatment; RCT: randomized controlled trial; Exp: experimental group; Con: control group; UE-Ex: upper extremity exercise; LE-Ex: lower extremity exercise; VO_2_: peak oxygen consumption.

**(d):** 

Study	Aim	Study design	Outcome measures	Findings	Conclusion
Barbeau and Visintin [45] Canada	Determine the level of transfer from treadmill training to overground locomotion and the factors most likely to impact locomotor recovery in chronic stroke patients	RCT (*n* = 100) Exp (*n* = 50): locomotor training + BWSTT Con (*n* = 50): locomotor training + full weight bearing Duration: 6 weeks of locomotor training	Overground walking Speed and endurance, functional balance, motor recovery Assessment: baseline, at the end of treatment	(i) Overground walking (+) (ii) Speed and endurance (+) (iii) Functional balance (+) (iv) Motor recovery (+)	The BWSTT training improved those with more severe gait impairments.
Duncan et al. [46] USA	To investigate if a systematic, progressive, physiologically based exercise program improves outcomes more than spontaneous recovery or standard treatment for subacute stroke	RCT (*n* = 92) Exp (*n* = 44): a progressive, structured, therapeutic exercise Con (*n* = 48): usual care Duration = 90 min/session, 36 sessions, 12-14 weeks	Balance (Berg and functional reach) Endurance (peak aerobic capacity and exercise duration) Mobility (timed 10 m walk and 6-minute walk distance) Assessment: baseline, 3 months	(i) BFR (+) (ii) Peak aerobic capacity and exercise duration (+) (iii) Timed 10 m walk and 6-minute walk distance (+)	A systematic, progressive program of therapeutic exercise resulted in improvements in endurance, balance, and mobility that were not the result of spontaneous recovery or routine care.
Pohl et al. [47] Germany	To evaluate the efficacy of BWSTT to limited progressive treadmill training (LTT) and conventional gait training (CGT) in subacute stroke patients	RCT (*n* = 60) Group 1 (*n* = 20): CGT Group 2 (*n* = 20): LTT Group 3 (*n* = 20): STT Duration: 12 sessions/4 weeks	Walking speed, cadence, stride length, functional ambulation category (FAC) Assessment: baseline, 2 weeks, end of study	(i) Walking speed (+) (ii) Cadence (+) (iii) Stride length (+) (iv) FAC (+)	BWSTT significantly improved poststroke walking ability compared to LTT or CGT.
Laufer et al. [48] Israel	To compare the effects of conventional overground gait training with treadmill training on subacute stroke patients	RCT (*n* = 25) Exp (*n* = 13): treadmill training Con (*n* = 12): overground training Five 30-minute gait training sessions each week for 15 weeks	Functional walking ability Walking speed Temporal characteristics of gait Assessment: baseline, at the end of treatment	Functional walking ability (+) Walking speed (+) Temporal characteristics of gait (+)	In the early phases of rehabilitation, stroke patients may tolerate treadmill exercise without a weight support device. Treadmill training improves walking function.

BWSTT: body weight-supported treadmill training; OGT: over gait training; CT: conventional treatment; RCT: randomized controlled trial; Exp: experimental group; Con: control group.

**(e):** 

Study	Aim	Study design	Outcome measures	Findings	Conclusion
Da Cunha Filho et al. [49] USA	To assess motor recovery between conventional therapy and conventional rehabilitation with BWSTT in a group of individuals with acute stroke	RCT (*n* = 12) Exp (*n* = 6): daily gait training on a treadmill with partial body weight support Con (*n* = 6): conventional therapy Five days a week till 2 to 3 weeks after completing the rehabilitation unit	Locomotor scale of the functional independence measure (FIM-L) VO_2_ Assessment pre/postintervention	FIM-L (−) VO_2_ (+)	Significant improvements in VO_2_ were seen in the BWSTT intervention group compared to the standard treatment group during cycling ergometry. There were no further substantial advantages in any physiologic or functional measures.
Nilsson et al. [50] Sweden	To compare the effects of BWSTT versus OGT in acute stroke patients	RCT (*n* = 73) Exp (*n* = 36): 30 minutes on a BWSTT, 5 days a week Con (*n* = 37): OGT	FIM Walking velocity for 10 m FAC Fugl-Meyer stroke assessment BBS Assessment: admission, discharge, and 10-month follow-up	FIM (−) Walking velocity for 10 m (−) FAC (−) Fugl-Meyer stroke assessment (−) BBS (−)	BWSTT is equivalent to OGT early in stroke rehabilitation.

BWSTT: body weight-supported treadmill training; OGT: over gait training; CT: conventional treatment; RCT: randomized controlled trial; Exp: experimental group; Con: control group; FIM: functional independence measure; FAC: functional ambulation classification; BBS: Berg balance scale.

## Data Availability

All data generated or analyzed during this study are included in this published article.
